# Radiological diagnostic accuracy study comparing Lenke, Bridwell, BSF, and CT-HU fusion grading scales for minimally invasive lumbar interbody fusion spine surgery and its correlation to clinical outcome

**DOI:** 10.1097/MD.0000000000019979

**Published:** 2020-05-22

**Authors:** José Antonio Soriano Sánchez, Sergio Soriano Solís, Manuel Eduardo Soto García, Héctor Antonio Soriano Solís, Briscia Yazmin Aranda Torres, José Alberto Israel Romero Rangel

**Affiliations:** a Spine Clinic of The American-British Cowdray Medical Center IAP. Campus Santa Fe; Mexican Association of Spine Surgeons (AMCICO); Mexican Society of Neurological Surgery (SMCN); Latin American Association of Neurosurgical Societies (FLANC).; b Orthopedic Surgeon and Minimally Invasive Spine Surgeon of The American-British Cowdray Medical Center IAP., Campus Santa Fe; c Neurosurgeon and Minimally Invasive Spine Surgeon of The American-British Cowdray Medical Center IAP., Campus Santa Fe; d Orthopedic Surgeon of The American-British Cowdray Medical Center IAP., Campus Santa Fe; e Student of Nutritional Sciences, University of Sonora (UNISON); Student of Professional Practices of The American-British Cowdray Medical Center IAP. Campus Santa Fe; f Neurosurgeon and Minimally Invasive Spine Surgeon of The American-British Cowdray Medical Center IAP. Campus Santa Fe; Neurosurgeon of the Regional General Hospital #25, Mexican Institute of Social Security (HGR#25, IMSS); Master in Sciences in Neurosurgery of the National Autonomous University of Mexico (UNAM).

**Keywords:** Bridwell, BSF, CT-HU, diagnostic accuracy study, Lenke, lumbar interbody fusion, minimally invasive spine surgery, pseudarthrosis, radiological lumbar interbody fusion grading scale, successful clinical outcome

## Abstract

Prospective randomized double-blinded diagnostic accuracy study about radiological grading of fusion after minimally invasive lumbar interbody fusion procedures (MI-LIFP).

To determinate the intra and the inter-observer correlation between different radiological lumbar interbody fusion grading scales (RLIFGS) in patients undergoing MI-LIFP and their correlation to clinical outcome.

Besides technological improvements in medical diagnosis and the many existing RLIFGS, surgical exploration continues to be the gold-standard to assess fusion in patients with radiological pseudarthrosis, with little if any research on the relationship between RLIFGS and clinical outcome.

We collected data from patients undergoing MI-LIFP procedures operated by a single surgeon from 2009 to 2017, which had follow-up and CT-scan control greater than 12 months, whose clinical registers specified lumbar and radicular visual analogue scale (L and R-VAS) and Oswestry Disability Index (ODI) score preoperatively and at the end of follow-up. Interbody fusion levels were coded for blinded evaluation by three different minimally invasive spine (MIS) surgeons, using Lenke, Bridwell, BSF (Brantigan, Steffe, Fraser), and CT-HU RLIFGS. We established fusion criteria, as described in their original papers. Another independent spine surgeon blindly evaluated successful clinical outcome (SCO), defined as a significant improvement in 2 of 3 of the following issues: L-VAS, R-VAS, or ODI score at follow-up; otherwise, rated as clinical pseudarthrosis. Radiological and clinical data was coded and statistically analyzed using Student T-Test, Pearson P-Test, and ANOVA with statistical package for the social sciences 21 by another blinded researcher, positive and negative predictive values were also calculated for each RLFGS.

We found a significant clinical improvement with a moderate intra-observer correlation between scales and no inter-observer or clinical correlation, with no sub-group statistically significant differences.

This paper represents the first study about the diagnostic accuracy of RLFGS, we concluded that their diagnostic accuracy is pretty low to determine fusion or pseudoarthrosis based on its low correlation to clinical outcome, we recommend surgeons rely on clinical findings to decide whether a patient has clinical fusion or pseudoarthrosis based on successful clinical outcome.

## Abstract 2

Supplemental Digital Content is available in the text

## 1 Introduction

Surgical treatment of the lumbar spine is effective to improve patient's pain, function, and disability with better cost-benefit performance relative to non-surgical treatment.^[1]^

Incidence of symptomatic pseudarthrosis after lumbar fusion procedures can be as high as 20%,^[2]^ accurate radiographic assessment is of paramount importance to identify patients who might benefit from additional surgery.^[3]^

Besides technological improvements in radiological diagnosis, surgeons have continued to struggle with imaging interpretation, many studies have outlined that to determine fusion status from x-rays or CT can be rather difficult, and methods vary widely across the literature^[4]^ radiographic criteria for fusion are often minimal, and probably underestimate pseudarthrosis rates.^[5]^ Improvement in computed tomography (CT) scan has increased accuracy in lumbar fusion assessment; several studies on helical CT have demonstrated high specificity for pseudarthrosis diagnosis compared with X-rays, particularly in the setting of lumbar interbody fusion surgery.^[3]^ There exist several radiological lumbar interbody fusion grading scales (RLIFGS), most of them like Lenke, Bridwell, and BSF with qualitative methods (also the most frequently used scales) and few ones using quantitative methods such as CT-HU score described by Ajler (2012), nevertheless, the “gold standard” for pseudarthrosis diagnosis remains surgical exploration.^[3]^

Furthermore, the diagnosis of pseudarthrosis is challenging, and no consensus on which clinical outcomes are needed to diagnose symptomatic pseudarthrosis; widely different criteria are used across literature, most of them dependent on untrusty and unvalidated methods. On the other hand, clinical research offers reliable and validated methods to measure improvements such as L-VAS, R-VAS, and ODI score, but its use continues to be limited and varies widely among series. Research comparing fusion is mostly confined to retrospective observational studies, and no single randomized clinical trial exists to evaluate the known and commonly used RLIFGS.^[6]^

Minimally invasive procedures have consistently contributed to shortening hospital stay and recovery time after lumbar interbody fusion procedures, suggesting satisfactory results, nevertheless, fully and internationally accepted criteria for clinical and radiological outcome success have not yet been published.

The present study aims to evaluate the diagnostic accuracy of existing RLIFGS through its intra and inter-observer correlation to grade fusion and its sensitivity and specificity to diagnose fusion based on its correlation to the definition of successful clinical outcome (SCO), a validated method for improved clinical outcome testing with simple and widespread used tools such as visual analogue scale for lumbar and radicular pain (L-VAS and R-VAS respectively) and Oswestry Disability Index (ODI) score.

## 2 Material and methods

### 2.1 Study design

Prospective randomized double-blinded diagnostic accuracy study (imaging studies interpretation and statistical analysis correlation) about radiological interpretation according to the Standards for Reporting of Diagnostic Accuracy Studies (STARD) statement, approved by the ethics and research hospital committee.

### 2.2 Participants

We collected data of postoperative CT scans performed to a cohort of patients undergoing minimally invasive lumbar spinal fusion procedures by a single surgeon (senior author, JASS) over 8 years from 2009 to 2017. We selected patient data that met the following criteria: follow-up and CT-scan control greater than 12 months, and whose registers specified L-VAS, R-VAS, and ODI score at preoperative and last follow-up.

### 2.3 Test method

Successful clinical outcome was defined as meeting 2 of either 3 of the following criteria: greater than 3 points drop in L-VAS, and R-VAS score or 30 points drop in ODI score from baseline to end of follow-up. Patient procedures were coded according to the level of interbody fusion as an independent case and randomized for blinded evaluation by three different minimally invasive spine surgeons previously trained in the proper use of each of the evaluated RLIFGS (Lenke, Bridwell, BSF, and CT-HU) by providing them with the original paper of the description.

### 2.4 Analysis

Resulting data and clinical outcomes were independently coded by a fourth minimally invasive spine surgeon and blindly analyzed by another researcher using Statistical Package for the Social Sciences 21 version. Lenke A and B, BSF 3 and Bridwell I and II were considered as evidence of fusion, and the remainder were considered as evidence of pseudarthrosis, as for CT-HU rating, values lower than 200 were considered pseudarthrosis and valued greater or equal to 200 were considered as fusion, as described by the original paper. We tested for a statistical significant clinical improvement in VAS and ODI score with Student T-Test, for intra and inter-observer correlation between RLIFGS and COS with Pearson P-Test, and ANOVA analysis to search for differences in fusion grading by age group, body mass index, comorbidities, level, and the number of interbody fusions by patient, and instrumentation technique (unilateral, mixed, or bilateral fixation), finally the sensitivity and specificity, as well as their positive and negative predictive values, were calculated.

## 3 Results

The summary of the result is shown in the STARD flow diagram (Appendix 1).

### 3.1 Participants

We identified 197 patients undergoing MI-LIFP from 2009 to 2017, 50 patients, 22 males (44%), and 28 females (56%) met criteria of inclusion (147 patients were excluded by having incomplete files). Mean age was 57.16 (SD 12.94, range 31–84), mean BMI was 25.19 (SD2.50, range 19.8–32), mean follow-up was 27.96 months (SD 14.28, range 12–69). Ten patients had diabetes mellitus as comorbidity, 9 had dyslipidemia, 8 had systemic hypertension, 8 had osteoporosis, and three had hypothyroidism. The main diagnosis was listhesis in 32 patients, scoliosis and listhesis in 6, 8 had disc degeneration, and facet arthropathy and 4 had listhesis with disc degeneration and facet arthropathy.

### 3.2 Test results

**Figure 1:**
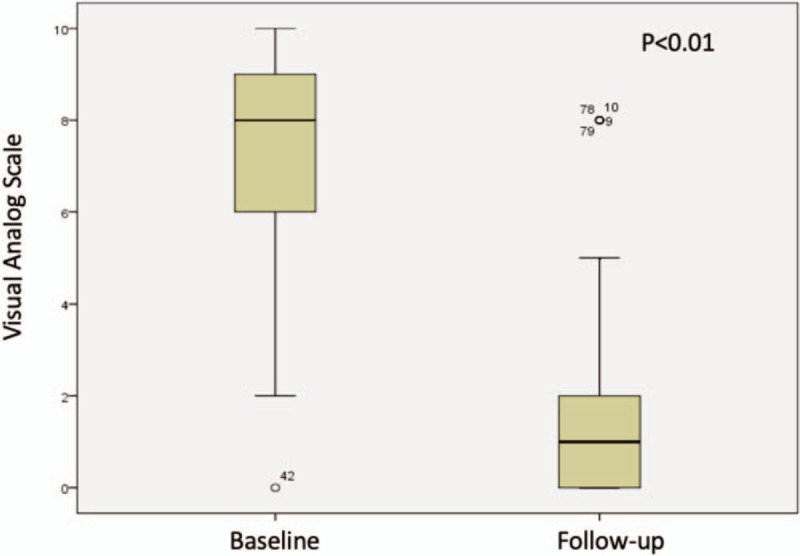
T-Student comparing lumbar VAS at baseline vs the end of follow-up.

**Figure 2:**
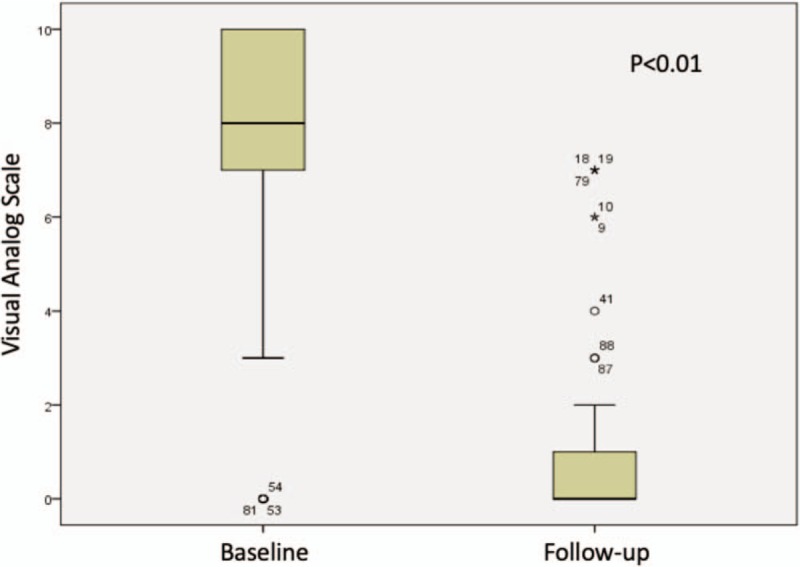
T-Student comparing radicular VAS at baseline vs the end of follow-up.

**Figure 3:**
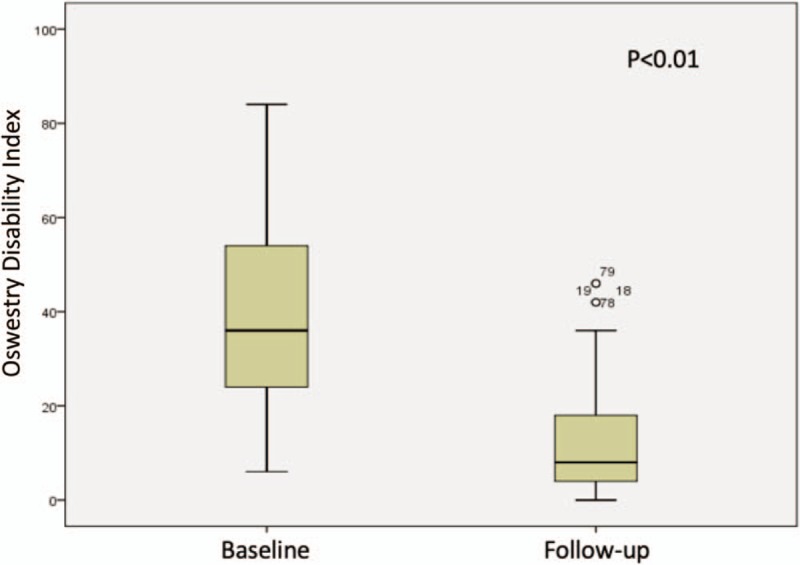
T-Student comparing Oswestry Disability Index at baseline vs the end of follow-up.

Mean baseline and final L-VAS was 7.26 (SD 2.35, range 0–10) and 1.11 (SD 1.98, range 0–8) respectively, mean R-VAS was 7.19 (SD 2.76, range 0–10) and 0.82 (SD 1.75, range 0–7) and mean ODI score was 37 (SD 18.75, range 6–84) and 11.12 (SD 10.86, range 0–46) respectively; statistical significant clinical improvement was seen in all of them by Student T-Test, Figures 1–3. Successful clinical outcome was determined in 41 patients (82%) with 75 levels, the rest nine patients (18%) with 15 levels had partial COS (at least one criteria with improvement and no other criteria with worst score), and no patient had worst clinical outcome (no improvement in any scale or even worst score at least in 1 criterion).

Ninety interbody fusion levels were studied, with a mean number of levels by patient of 1.78 (SD 0.84, range 1–4), most patients had 2 level surgery (25 patients 50%), L4-L5 and L5-S1 were the most commonly involved segments in 20 patients (40%) and the segment most frequently affected as a single-level disease was L5-S1 in 11 patients (22%). Table 1 shows fusion grading according to individual observers by specific RLFGS. Mean fusion rates, as determined by all three observers by each RLIFGS, are shown in Table 2.

**Table 1:**
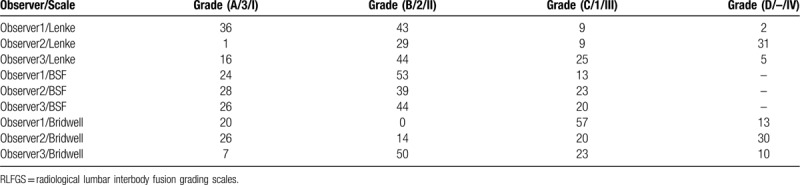
Fusion grading according to individual observer by specific RLIFGS.

**Table 2:**
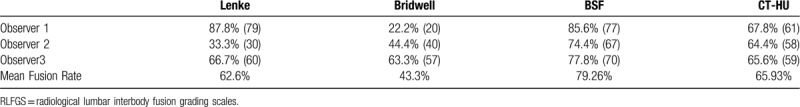
Mean fusion rate as determined by all three observers by RLIFGS.

CT-HU values for each observer were: observer1 234.34 Mean CT-HU value (range 218.30–250.38 95% confidence interval, CI), observer2 228.58 Mean CT-HU value (range 210.86–246.3195% CI), observer3 234.23 Mean CT-HU value (range 215.78–252.67, 95% CI).

Intra-observer evaluation correlation by observer1 were 0.602 (*P* < .01) for Lenke/Bridwell, 0.639 (*P* < .01) for Lenke/BSF, 0.110 for Lenke/CT-HU, 0.685 (*P* < .01) for Bridwell/BSF, −0.103 for Bridwell/CT-HU and 0.067 for BSF/CT-HU

Intra-observer evaluation correlation by observer2 were 0.789 (*P* < .01) for Lenke/Bridwell, 0.825 (*P* < .01) for Lenke/BSF, 0.063 for Lenke/CT-HU, 0.685 (*P* < .01) for Bridwell/BSF, 0.118 for Bridwell/CT-HU and −0.012 for BSF/CT-HU

Intra-observer evaluation correlation by observer3 were 0.505 (*P* < .01) for Lenke/Bridwell, 0.535 (*P* < .01) for Lenke/BSF, −0.093 for Lenke/CT-HU, 0.026 (*P* < .01) for Bridwell/BSF, −0.044 for Bridwell/CT-HU and −0.012 for BSF/CT-HU

Inter-observer evaluation correlations by Lenke RLFGS were, 0.291 (*P* < .01) for observer1/observer2, 0.248 (*P* < .01) for observer1/observer3 and 0.315 (*P* < .01) for observer2/observer3.

Inter-observer evaluation correlations by Bridwell RLFGS were, 0.246 (*P* < .01) for observer1/observer2, 0.346 (*P* < .01) for observer1/observer3 and 0.341 (*P* < .01) for observer2/observer3

Inter-observer evaluation correlations by BSF RLIFGS were, 0.197 for observer1/observer2, 0.329 (*P* < .01) for observer1/observer3 and 0.263 (*P* < .01) for observer2/observer3

Inter-observer evaluation correlations by CT-HU RLIFGS were, 0.910 (*P* < .01) for observer1/observer2, 0.862 (*P* < .01) for observer1/observer3 and 0.943 (*P* < .01) for observer2/observer3

Correlations between the attained grade of fusion by RLIFGS and COS for observer1 are 0.015 for Lenke, 0.096 for Bridwell, −0.014 for BSF, and 0.011 for CT-HU none of them statistically significant correlations.

Correlations between the attained grade of fusion by RLIFGS and COS for observer2 are −0.126 for Lenke, -0.020 for Bridwell, −0.262 for BSF and −0.083 for CT-HU none of them statistically significant correlations.

Correlations between the attained grade of fusion by RLIFGS and COS for observer3 are 0.000 for Lenke, -0.155 for Bridwell, −0.096 for BSF and −0.136 for CT-HU none of them statistically significant correlations.

Correlations between the overall (mean value for 3 observers) attained grade of fusion by RLFGS and COS are −0.118 for Lenke, −0.059 for Bridwell, −0.119 for BSF and −0.046 for CT-HU, none of them statistically significant correlations.

ANOVA sub-group tests with post hoc test by Tukey-B showed a statistically significant difference with better fusion for 3 level surgery graded by Lenke, Bridwell, and BSF but not to CT-HU with a (*P* < .05) for observer1

ANOVA sub-group tests with post hoc test by Tukey-B showed no statistically significant differences by observer2

ANOVA sub-group tests with post hoc test by Tukey-B showed a statistically significant difference with a better grade of fusion attained by left-unilateral instrumentation by BSF with a *P* < .05 and the worst fusion attained by Bridwell with a *P* < .05 for hypertension and diabetes mellitus comorbidities for observer3

ANOVA sub-group tests with post hoc test by Tukey-B showed a statistically significant difference with better fusion for hypertension comorbidity attained by BSF with a *P* < .05 for overall score (mean of 3 observers)

Table 3 summarizes overall sensitivity, specificity, positive, and negative predictive value for fusion and non-fusion (symptomatic pseudarthrosis) diagnosis by each RLFGS.

**Table 3:**
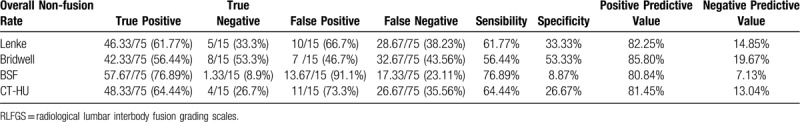
Overall sensitivity, specificity, positive and negative predictive value for fusion and non-fusion (symptomatic pseudarthrosis) diagnosis by each RLIFGS.

**Figure 4:**
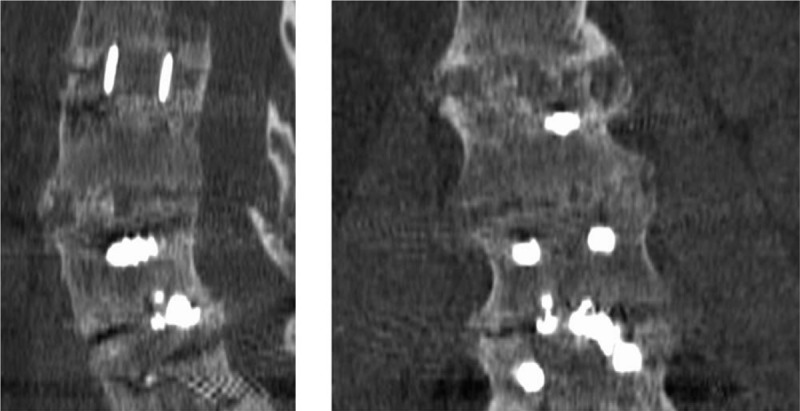
Example of non-agreement for inter-observer grading to the L3-L4 lumbar interbody fusion procedure (Image Center). Observer1: Bridwell I, BSF 3, Lenke A; observer2 Bridwell III, BSF 2, Lenke A, observer3: Bridwell IV, BSF 1, Lenke B. It demonstrates the moderate intra-observer correlation and the lack of inter-observer correlation.

Figure 4 shows a relevant CT-scan evaluation as an example of grading by observers.

## 4 Discussion

A good intra-observer correlation exists between BSF, Bridwell, and Lenke, but not to CT-HU. A good inter-observer correlation exists between the CT-HU scale but not for BSF, Bridwell, and Lenke. A correlation does not exist between a successful clinical outcome by L-VAS and R-VAS and Oswestry Disability Index and a good grade of fusion by the different RLFGS. Our results confirm our initial hypothesis; RLFGS do not reflect clinical outcomes and are not a fair tool to evaluate clinical fusion nor to discern clinical pseudoarthrosis. Based on these results, we suggest relaying on clinical findings to determine whether a patient is clinically fused or has clinical pseudoarthrosis. We agree with other authors that besides the impressive advances in radiological assessment and diagnosis, currently there does not exist any reliable radiological test or scale to diagnose pseudarthrosis other than the surgical exploration, which currently represents the gold-standard for symptomatic pseudarthrosis. Besides, most spine surgeons may prefer to trust clinical evaluation scales such as L/R-VAS and ODI as a means to diagnose symptomatic pseudarthrosis. Table 4 shows the present study results as compared to other current clinical series evaluating fusion and symptomatic pseudarthrosis rates, as determined by clinical outcome scales, such as ODI and L/R-VAS. We obtained symptomatic improvement in 100% of cases, with a rate as low as 4% of symptomatic pseudarthrosis in patients whom despite a symptomatic improvement, continue to have any residual lumbar pain in the presence of radiological non-fusion as evaluated by the different RLIFGS, the present work represents the only one minimally invasive randomized, double-blind clinical trial available evaluating fusion with available RLIFGS correlate to internationally accepted quantitative parameters that successful clinical outcome criteria.^[3–5,11–16]^

**Table 4:**
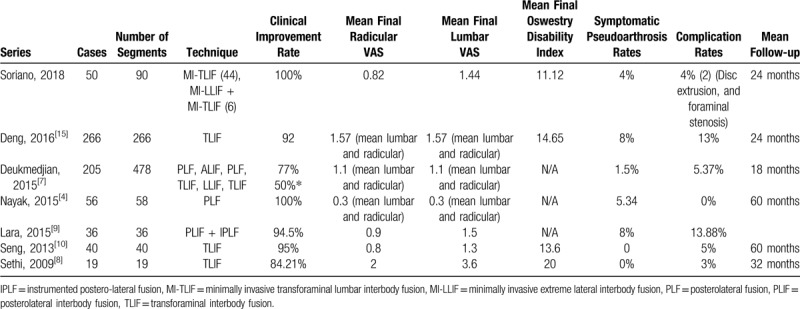
Comparison to other current clinical series evaluating fusion and symptomatic pseudarthrosis rates by successful clinical outcome parameters.

## 5 Conclusions

All of the RLIFGS (except CT-HU) measure qualitative parameters (graft resorption and remodeling, radiolucency, and construct collapse); many of them are confusing, misinterpreted, or mutually excluding in the same classification, any of these circumstances may contribute to the low inter-observer correlations. CT-HU could be a better tool for assessing bone formation; nevertheless, the use of any RLIFGS should be avoided to assess clinical fusion or pseudarthrosis due to a low to absent correlation to clinical outcome. This paper represents the first study about the diagnostic accuracy of RLFGS to determine clinical fusion or pseudoarthrosis; we concluded that their diagnostic accuracy is pretty low to determine fusion or pseudoarthrosis based on its low correlation to clinical outcome. We recommend surgeons rely on clinical findings to decide whether a patient has clinical fusion or pseudoarthrosis based on a successful clinical outcome (as defined by a significant improvement in 2 of 3 of the following factors: radicular and axial VAS, and ODI score) instead of radiological fusion by RLFGS).

## Acknowledgments

The authors thank their families and friends for the time and support given over all these years.

## Author contributions

**Conceptualization:** José Alberto Israel Romero Rangel.

**Data curation:** José Alberto Israel Romero Rangel, Sergio Soriano Solís, Manuel Eduardo Soto García, Héctor Antonio Soriano Solís, Briscia Yazmin Aranda Torres.

**Formal analysis:** José Alberto Israel Romero Rangel.

**Funding acquisition:** José Antonio Soriano Sánchez.

**Investigation:** José Alberto Israel Romero Rangel, Manuel Eduardo Soto García.

**Methodology:** José Alberto Israel Romero Rangel.

**Project administration:** José Alberto Israel Romero Rangel.

**Resources:** José Antonio Soriano Sánchez.

**Software:** José Antonio Soriano Sánchez.

**Supervision:** José Alberto Israel Romero Rangel, José Antonio Soriano Sánchez.

**Validation:** José Alberto Israel Romero Rangel, Sergio Soriano Solís, Manuel Eduardo Soto García, Héctor Antonio Soriano Solís, Briscia Yazmin Aranda Torres, José Antonio Soriano Sánchez.

**Visualization:** José Alberto Israel Romero Rangel.

**Writing – original draft:** José Alberto Israel Romero Rangel, Briscia Yazmin Aranda Torres.

**Writing – review & editing:** José Alberto Israel Romero Rangel, José Antonio Soriano Sánchez.

## Supplementary Material

Supplemental Digital Content
