# Supplementary figures and images for: Radiological diagnostic accuracy study comparing Lenke, Bridwell, BSF, and CT-HU fusion grading scales for minimally invasive lumbar interbody fusion spine surgery and its correlation to clinical outcome
Source: Medicine (Baltimore). 2020 May 22;99(21):e19979. doi: 10.1097/MD.0000000000019979 (PMC7250056; doi:10.1097/MD.0000000000019979)

Appendix 1. STARD flow diagram of study

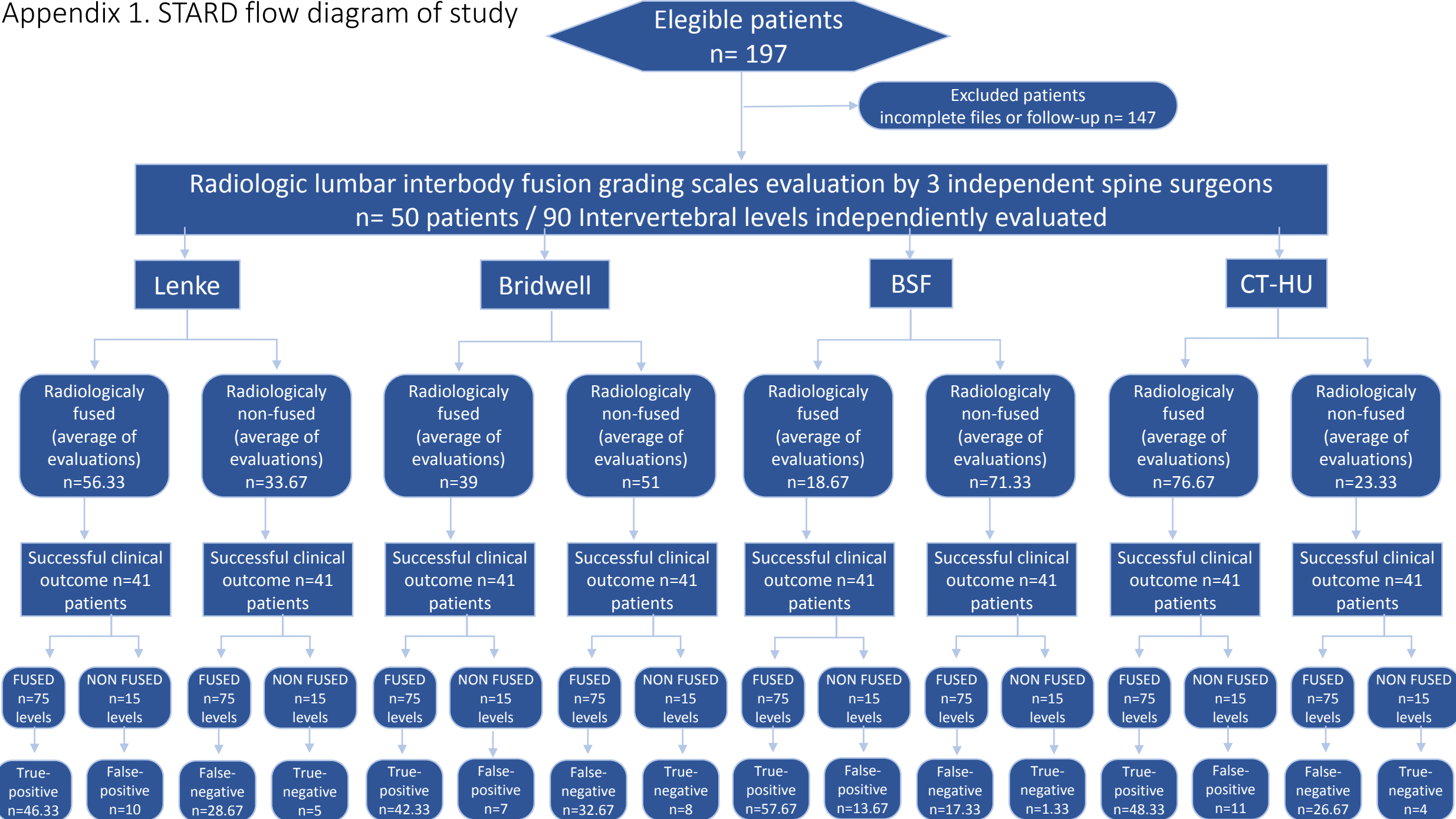

Supplement: Supplemental Digital Content [file medi-99-e19979-s001.pdf]
